# Experimental studies of thermal performance of an evacuated tube heat pipe solar collector in Polish climatic conditions

**DOI:** 10.1007/s11356-020-07920-3

**Published:** 2020-02-12

**Authors:** Alicja Siuta-Olcha, Tomasz Cholewa, Kinga Dopieralska-Howoruszko

**Affiliations:** Faculty of Environmental Engineering, Lublin University of Technology, Lublin, Poland

**Keywords:** Solar energy, Evacuated tube solar collector, Thermal efficiency, Exergy efficiency

## Abstract

This work presents the results of experimental studies on the energy performance of an evacuated solar collector, heat pipe type, consisting of 24 tubes, over the period of 2 months. The solar collector with a gross area of 3.9 m^2^ is part the solar hot water test system located in Lublin (Poland). The effect of the weather conditions and operating parameters on the thermal and exergy efficiencies of the evacuated tube solar collector has been defined. The solar irradiation per month for July amounted to 80 kWh/m^2^, and for August, it equalled 112.8 kWh/m^2^. The average thermal gain was found to be in July 163 W/m^2^ and in August 145 W/m^2^, respectively. For the considered study period, the average value of energy yield in the solar collector was obtained at the level of 4.28 MJ/(m^2^·d). The average monthly energy efficiencies of the solar collector in July and August were 45.3% and 32.9%, respectively, while the average monthly exergy efficiencies reached 2.62% and 2.15%, respectively. Increasing the wind speed to 0.86 m/s decreases the thermal efficiency and the exergy efficiency by 67% and 41%, respectively.

## Introduction

One of the most important ways to protect the natural environment is the gradual introduction of the energy-saving technologies into the industry and the renewable energy sources in the energy sector – instead of the conventional energy sources. The use of solar energy allows reducing both the consumption of fossil fuels and the resultant emission of harmful pollutants introduced into the atmosphere. The thermal conversion of solar radiation energy does not contaminate the natural environment. The limitation in its use may be the uncertainty and periodicity of energy supply in time due to the geographical location and climate.

The use of 1 m^2^ of solar collector area allows reducing the consumption of 250 kg of coal during the year, thus reducing the emission of particulate matter by 25 kg, sulfur dioxide by 6 kg, and nitrogen oxides by 2 kg. The total capacity in operation of solar heating systems installed in the world in 2017 was 472 GW_th_, which corresponded to 675 million square meters of solar collectors. The annual solar thermal energy yield in 2017 amounted to 388 TW·h, which translates to savings of 41.7 million tons of oil and 134.7 million tons of CO_2_ (Weiss and Spörk-Dür 2018).

The practical application of solar energy is varied and includes domestic hot water preparation, swimming pool heating, space heating, water and food preparation for animals in farm buildings, drying of agricultural products, solar district heating, solar air conditioning and cooling, desalination of sea water or discharge water, and heating of industrial processes (Zambolin and Del Col 2010; Sabiha et al. 2015; Kumar et al. 2017; Elsheniti et al. 2019; Naik et al. 2019; Siuta-Olcha et al. 2019).

Solar collectors are used to obtain solar energy, convert this energy into heat, and then transfer heat to the flowing working medium (Chwieduk 2011; Kalogirou et al. 2016). Evacuated tube solar collectors function effectively even in low sunlight and at low outdoor air temperatures. On the other hand, the working medium in the evacuated collector can reach high temperatures in the range of 50–200 °C (Sharma and Diaz 2011; Sabiha et al. 2015; Chopra et al. 2018; Saxena and Gaur 2018).

In Poland, the average annual irradiation is in the range of 95–1150 kWh/(m^2^·year), and the average value of sunshine hours is 1802 h/year. The share of solar energy in all renewable energy sources was set at 0.58%, based on the 2016 data. In majority of cases, these are solar pumping systems for domestic hot water preparation and space heating in residential and public buildings (hotels, hospitals, schools). The total installed capacity in Polish solar installations with flat and evacuated solar collectors was at the level of 1496.1 MW_th_. In Poland, 2,137,200 m^2^ of solar collectors were installed, 77.7% of which corresponded to the flat solar collectors while 22.3% to the evacuated solar collectors. The calculated annual collector yield is equal to 873 GWh/year, which corresponds to energy savings at the level of 93,802 t_oe_/year and CO_2_ reduction at 302,794 t_CO2_/year (Weiss and Spörk-Dür 2018).

Ayompe et al. (2011) conducted an energy and economic analysis of two solar hot water systems with flat plate and evacuated tube collectors situated in Dublin, Ireland, based on annual experimental studies. The flat plate solar collectors with an aperture area of 4 m^2^ generated 496-kWh/m^2^ energy per unit area, and the evacuated solar collector type heat pipe with an absorber area of 3 m^2^ generated 681 kWh/m^2^. The average seasonal thermal efficiency of the evacuated tube collector ranged from 51.5 (winter) to 65.6% (spring). The average annual efficiency of the evacuated solar collector was determined at 60.7%. On the basis of the continuing experimental investigations, an average annual value of thermal efficiency of the evacuated tube solar collector was obtained, equal to 63.2% (Ayompe and Duffy 2013).

Hayek et al. (2011) conducted experimental studies for the thermal performance of two types of evacuated solar collectors, i.e., a heat pipe and water-in-glass for a period of 3 months under the climate conditions of Zouk Mosbeh, Lebanon. On the basis of these investigations, they found that the energy efficiency of the heat pipe collectors is definitely higher than the efficiency of the water-in-glass collectors (from 15 to 20%) and the slope of the collector surface does not have a significant impact on its thermal efficiency.

Nkwetta et al. (2013) performed experiments on four types of evacuated and non-evacuated internal compound parabolic concentrating solar collectors in a test installation to determine and compare their optical efficiencies, heat loss coefficients, and total energy collected.

Tong et al. (2016) performed simulation and experimental studies on the thermal performance of a concentric evacuated tube collector. The experiment was conducted in Gwangju, Korea. The solar collector consisted of 20 tubes and was inclined at an angle of 45°. The mass flow rate of the solar fluid in the form of a water-propylene glycol mixture was kept at the level of 0.065 kg/s. The verified model was used to simulate the thermal efficiency of the solar collector for four locations (Gwangju, Jeju, Seoul, Daejeon) and for different tilt angles of the solar collector, with special consideration of 3 months of the winter season.

Daghigh and Shafieian (2016) presented the thermal energy and exergy balance equations for an evacuated tube solar collector. The MATLAB environment was used to conduct theoretical analyses and simulations of the thermal efficiency of the solar hot water installation. The mathematical model was verified. The test solar system was located in Sanandaj, Iran. It was found that the exergy efficiency of the solar collector with an absorber area of 2.06 m^2^ usually increased in the evening (6:00 PM) and reached the maximum value of about 4.5%. The analyses took into account the consumption of hot water at particular hours of the day. It was found that there is a need to optimize the mass flow rate of the solar fluid in order to achieve high thermal efficiency.

Similar studies were conducted for a solar installation with a solar collector type heat pipe with a gross area of 3.93 m^2^ located in Perth, Australia (Shafieian et al. 2019).

Maraj et al. (2019) presented the results of a detailed analysis of the work of the solar water heating system with an evacuated tube collector type heat pipe, located in Tirana, Albania. The monthly and annual values of energy yields from the solar collector, useful energy delivered to the storage tank, and heat losses from the solar collector, from the collector circuit, and from the storage tank were provided. The collector’s annual thermal efficiency of 62% was obtained.

Elsheniti et al. (2019) tested the thermal efficiency of a heat pipe evacuated tube solar collector operating in solar absorption cooling systems under the weather conditions of Alexandria, Egypt, both theoretically and experimentally.

Krawczyk et al. (2019) conducted the comparative studies of the thermal efficiency of solar collectors in a hot water installation in an office building for three locations: Bialystok, Cordoba, and Kaunas.

Allouhi et al. (2019) conducted the simulation studies of energy yields of a solar installation with a heat pipe flat plate collector with a gross area of 2.27 m^2^ at the inclination of 30°, located in Fez, Morocco. The investigations were carried out for January using the MATLAB software. It was found that the instantaneous energy efficiency reached a maximum value of 55% and the exergy efficiency of 6.9%.

This paper examines both thermal and exergy efficiencies of a heat pipe evacuated tube solar collector located in Lublin, Poland, based on the experimental data for two summer months.

## Materials and methods

### Description of an active solar hot water test system

**Fig. 1:**
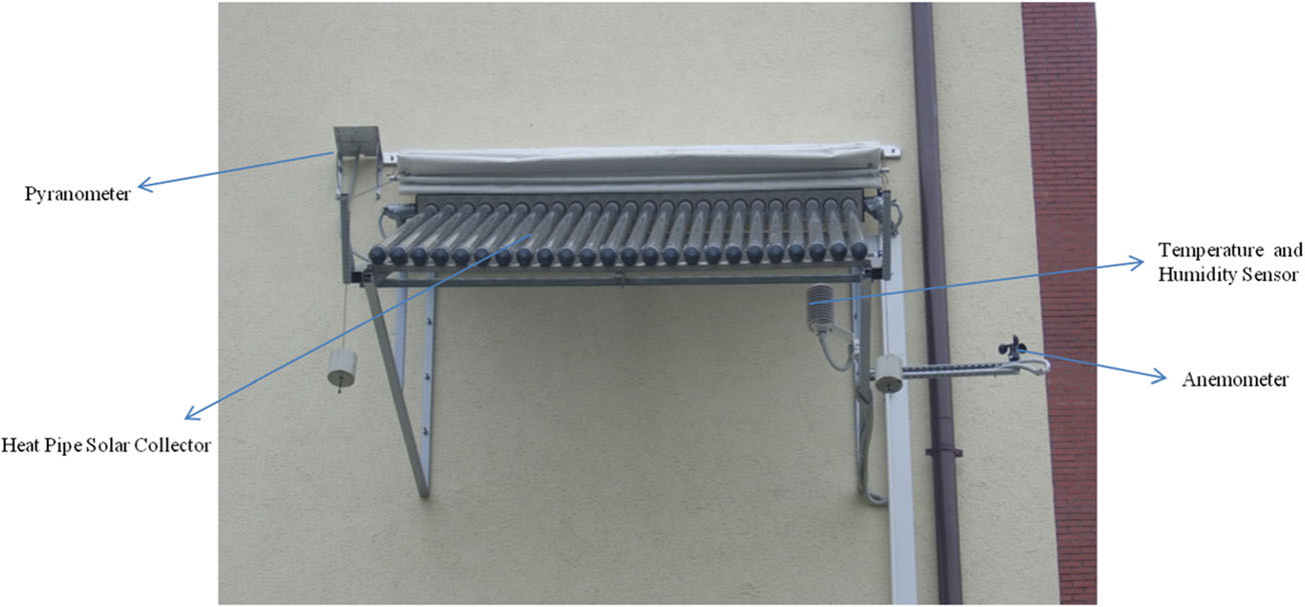
Analyzed heat pipe evacuated tube solar collector with a weather station

The solar hot water test system is located in the laboratory of the Faculty of Environmental Engineering, Lublin University of Technology. Lublin is a city situated in the eastern part of Poland, and its geographic coordinates are 51°15′N (latitude) and 22°34′E (longitude). Lublin has a humid continental climate (it is Dfb zone according to the Köppen-Geiger climate classification). The Solarglas SG1800/24 heat pipe evacuated tube solar collector (Fig. 1) is mounted on the southeastern facade of the building of the Faculty, on the assembly frame at an angle of 38° to the level. The main technical specifications of the solar collector are presented in Table 1.

**Table 1:** Technical data of the heat pipe evacuated tube solar collector

Parameter	Value
Aperture area	3.6 m^2^
Gross area	3.9 m^2^
External dimensions	2.040 *×* 1.994 *×* 0.157 m
Number of evacuated tubes	24
Absorptivity coefficient of the absorber	95%
Emissivity coefficient of the absorber	5%
Vacuum tube material	Borosilicate glass
Glass thickness	0.0016 m
Outer/inner diameter of a tube	0.058 m/0.047 m
Absorption coating	Al/N/Al
Insulation thickness of a manifold	0.055 m
Stagnation temperature	≥ 523 K
Gross weight (empty)	80 kg
Solar fluid content	1.6·10^−3^ m^3^
Maximum operating pressure	6 bar
Hydraulic connection/outer diameter	copper tube/0.022 m

The hot water storage tank is a device with a total capacity of 0.35 m^3^, 1.87 m height with a diameter of 0.50 m, and a maximum operating pressure of 3 bar. It was made of steel and was thermally insulated by means of mineral wool with a thickness of 0.10 m and covered with an outer jacket made of galvanized steel sheet. Inside the tank, in its lower part, there is a spiral steel heating coil with a surface area of 1.5 m^2^, diameter of 0.0269 × 0.0023 m, and 18 m in length. The solar installation was made of thermally insulated copper pipes. A mixture of water and propylene glycol with the concentration of 50% was used as a working fluid in a solar collector circuit.

The solar system is protected against the pressure increase with a safety valve and a diaphragm expansion vessel with a capacity of 0.018 m^3^ and with a maximum operating pressure of 6 bar. The flow of the working medium in the solar circuit is forced by the Wilo Stratos 25/1-6 pump with adjustable capacity. The flow control range on the rotameter ranges from 1.67·10^−5^ to 21.67·10^−5^ m^3^/s. The solar installation controller type Digisol maxi plus is built into the hydraulic group.

### Description of a measurement system

Two interfaces were used to log and store data every 5 min. The applied measurement system allows continuous measurement of meteorological parameters (the AL154SAVDA5.6U.1L interface), parameters of the test installation at characteristic points (the AL154M1SAVDA5 interface) as well as programmable control of the operation of the circulating solar pump, and the bleed valve located in the hot water pipe.

The AL154SAVDA5.6U.1L interface was used to measure the following parameters: internal temperature of the laboratory room, temperature and relative humidity of the outdoor air, solar radiation intensity, and wind speed. The calibrated PT500 platinum resistance temperature sensors were used to carry out the measurements of the working fluid temperature in the collector’s circulation and of water in the tank. The solar installation was equipped with the LQM-III heat meter. The rated accuracy of the sensors was presented in Table 2 (Siuta-Olcha 2012; Siuta-Olcha et al. 2019).

**Table 2:** Accuracy of sensors used to measure the weather parameters and temperature of a working fluid and water

Sensor/parameter	Accuracy
Rotronic HygroClip/*T*_*a*_, *φ*_*a*_	At 296 K: ± 1% rh/± 0.3 K
Kipp&Zonnen Pyranometer CMP 6/*I*_*sol*_	ISO classification: first classDirectional error (at 353 K and 1000 W/m^2^): ± 20 W/m^2^ Sensitivity: 15.40 μV/W/m^2^
Cup anemometer/*v*_*w*_	± 0.5 m/s, measuring range: 0–50 m/s
Temperature sensor PT500/*T*_*f,*1_, *T*_*f,*2_	Accuracy class A, at 323 K: ± 0.25 K

### Uncertainty analysis

The uncertainty of the measured parameters depends primarily on random errors, individual instrument accuracy, instance calibration errors, and data acquisition errors (Shafieian et al. 2019; Sharafeldin et al. 2019). The average relative error was calculated for such parameters as

useful heat gain,

1 $$ {\dot{Q}}_u=\mathrm{f}\left({\dot{V}}_{col},{\rho}_{W- PG},{C}_{p,W- PG},{T}_{f,2},{T}_{f,1}\right) $$

thermal efficiency,

2 $$ {\eta}_{TH}=\mathrm{f}\left({\dot{V}}_{col},{\rho}_{W- PG},{c}_{p,W- PG},{T}_{f,2},{T}_{f,1},{I}_{sol}\right) $$

and exergy efficiency

3 $$ {\eta}_{EX}=\mathrm{f}\left({\dot{V}}_{col},{\rho}_{W- PG},{c}_{p,W- PG},{T}_{f,2},{T}_{f,1},{I}_{sol},{T}_a\right) $$

using the following equations:

4 $$ {\delta}_{{\dot{Q}}_u}\left(\%\right)={\left[{\delta}_{{\dot{V}}_{col}}^2+{\delta}_{\rho_{W- PG}}^2+{\delta}_{c_{p,W- PG}}^2+{\left(\frac{\Delta _{T_{f,1}}}{T_{f,1}}\right)}^2+{\left(\frac{\Delta _{T_{f,2}}}{T_{f,2}}\right)}^2\right]}^{1/2}\bullet 100\% $$

5 $$ {\delta}_{\upeta_{TH}}\left(\%\right)={\left[{\delta}_{{\dot{V}}_{col}}^2+{\delta}_{\rho_{W- PG}}^2+{\delta}_{c_{p,W- PG}}^2+{\left(\frac{\Delta _{T_{f,1}}}{T_{f,1}}\right)}^2+{\left(\frac{\Delta _{T_{f,2}}}{T_{f,2}}\right)}^2+{\left(\frac{\Delta _{I_{sol}}}{I_{sol}}\right)}^2\right]}^{1/2}\bullet 100\% $$

6 $$ {\delta}_{\upeta_{EX}}\left(\%\right)={\left[{\delta}_{{\dot{V}}_{col}}^2+{\delta}_{\rho_{W- PG}}^2+{\delta}_{c_{p,W- PG}}^2+{\left(\frac{\Delta _{T_{f,1}}}{T_{f,1}}\right)}^2+{\left(\frac{\Delta _{T_{f,2}}}{T_{f,2}}\right)}^2+{\left(\frac{\Delta _{I_{sol}}}{I_{sol}}\right)}^2+{\left(\frac{\Delta _{T_a}}{T_a}\right)}^2\right]}^{1/2}\bullet 100\% $$

The uncertainties in measurement were set at 1.4% for the useful heat gain, 4.3% for thermal efficiency, and 4.5% for exergy efficiency.

### Research methodology

The obtained measurement data, registered every 5 min by the measuring system of the research installation, enabled a detailed analysis of thermal and exergy efficiencies of the heat pipe evacuated tube solar collector. The employed measurement system software, enabled to collect and save data for each day in separate *xlsx* files as well as conduct visualization of data. For detailed calculations and analysis, the values of recorded measurement data were averaged for each hour of the day.

The solar irradiation per day (*SID*) is defined as:

7 $$ SID\left(\frac{kW\bullet h}{m^2\bullet d}\right)=\frac{\sum \limits_{\mathrm{i}=1}^{\mathrm{n}}\left({I}_{sol,i}\bullet {\mathrm{t}}_{\mathrm{p}}\right)}{3600000} $$

where *n* is number of measurement periods for a given day, *I*_*sol*, *i*_ is a mean value of total solar irradiance in W/m^2^, and t_p_ is the duration of the measurement period in *s*.

The energy yields of the solar collector depend on both the weather conditions: the external temperature, insolation, and the solar fluid temperature at the collector inlet (Joo and Kwak 2017).

The instantaneous energy efficiency of a solar collector is dependent on the useful heat gain, the solar irradiance, and the collector area. On the basis of the first law of thermodynamics, the thermal efficiency of the solar collector is given by the Eq. (8) (Ataee and Ameri 2015; Alfaro-Ayala et al. 2015; Sabiha et al. 2015; Kalogirou et al. 2016; Saxena and Gaur 2018; Kaya et al. 2018):

8 $$ {\upeta}_{TH}=\frac{{\dot{Q}}_u}{I_{sol}\bullet {A}_{col}}\kern0.5em $$

The useful heat gain (${\dot{Q}}_u$) to the working fluid is calculated as:

9 $$ {\dot{Q}}_u(W)={\dot{m}}_{col}\bullet {c}_{p,W- PG}\bullet \left({T}_{f,2}-{T}_{f,1}\right) $$

The thermal efficiency of the solar collector can also be expressed as an equation (Kaya and Arslan 2019):

10 $$ {\upeta}_{TH}={F}_R\left(\tau \bullet \alpha \right)-{F}_R{U}_L\frac{\left({T}_{f,1}-{T}_a\right)}{I_{sol}} $$

The thermal efficiency of the heat pipe evacuated tube solar collector was calculated for the experimental data. The working fluid employed is a mixture of water and propylene glycol with the concentration of 50%. Specific heat capacity and density of this fluid were determined on the basis of the formulas given in (Chekerovska and Filkoski 2015):

11 $$ {c}_{p,W- PG}\ \left(\frac{J}{kg\bullet K}\right)=3901-2.674\bullet {T}_{f,m}+0.0058\bullet {T}_{f,m}^2+5\bullet {10}^{-8}\bullet {T}_{f,m}^3 $$

12 $$ {\rho}_{W- PG}\ \left(\frac{kg}{m^3}\right)=978.2+0.973\bullet {T}_{f,m}-0.003\bullet {T}_{f,m}^2+1\bullet {10}^{-6}\bullet {T}_{f,m}^3 $$

The analysis was performed by taking into account the constant working fluid flow rate through the collector at the level of 0.055 kg/s and assuming zero pressure drop in the collector.

Energy is based on the first law of thermodynamics and can only be used to determine the quantity of energy. Exergy is based on the second law of thermodynamics; it represents the quality of energy and involves the irreversibility while analyzing solar collector efficiency (Park et al. 2014). Conducting the energy analysis does not provide the information on the occurrence of internal process losses (Petela 2017). In contrast to energy, exergy is not subject to the law of conservation; it provides the information whether there is a possibility to improve the process, i.e., to reduce losses of exergy. Exergy is defined as the maximum ability of the considered portion of energy to perform work using the heat taken from the environment as well as the commonly occurring and mutually independent components of the environment (Szargut and Petela 1965; Farahat et al. 2009; Kalogirou et al. 2016).

The exergy efficiency of the solar collector is defined as the ratio between the increase of the fluid exergy rate and the exergy of solar radiation (Farahat et al. 2009; Kalogirou et al. 2016) and can be written as:

12 $$ {\upeta}_{EX}=\frac{\dot{E}{x}_{f,2}-\dot{E}{x}_{f,1}}{\dot{E}{x}_{1,S}} $$

The outlet exergy rate includes only the exergy rate of outlet fluid flow and is calculated from:

13 $$ \dot{E}{x}_{f,2}(W)={\dot{m}}_{col}\bullet {c}_{p,W- PG}\bullet \left({T}_{f,2}-{T}_a-{T}_a\bullet \mathit{\ln}\left(\frac{T_{f,2}}{T_a}\right)\right) $$

The inlet exergy rate with fluid flow is given by:

14 $$ \dot{E}{x}_{f,1}(W)={\dot{m}}_{col}\bullet {c}_{p,W- PG}\bullet \left({T}_{f,1}-{T}_a-{T}_a\bullet \mathit{\ln}\left(\frac{T_{f,1}}{T_a}\right)\right) $$

The total inlet exergy by solar radiation is obtained from:

15 $$ \dot{E}{x}_{1,S}(W)={I}_{sol}\bullet {A}_{col}\bullet \left(1-\frac{T_a}{T_S}\right) $$

Using Eqs. (12)–(15), the exergy efficiency of the collector is given by (Jafarkazemi and Ahmadifard 2013; Jafarkazemi et al. 2016; Siuta-Olcha et al. 2019):

16 $$ {\upeta}_{EX}=\frac{{\dot{m}}_{col}\bullet {c}_{p,W- PG}\bullet \left[\left({T}_{f,2}-{T}_{f,1}\right)-{T}_a\left(\mathit{\ln}\frac{T_{f,2}}{T_{f,1}}\right)\right]}{I_{sol}\bullet {A}_{col}\left[1-\left(\frac{T_a}{T_s}\right)\right]} $$

where *T*_*s*_ is the apparent sun temperature in *K* (*T*_*s*_=4500 K) (Petela 1964; Farahat et al. 2009; Jafarkazemi and Ahmadifard 2013; Jafarkazemi et al. 2016).

## Results and discussion

**Fig. 2:**
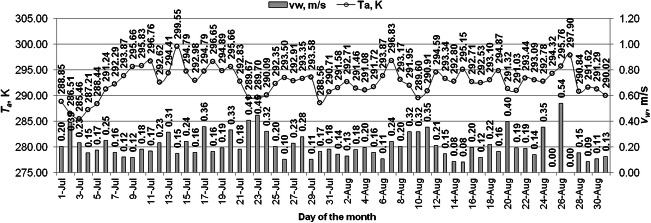
Ambient temperature and wind velocity variations in considered period of studies

The analysis was carried out for 2 months, i.e., for July and August. The courses of changes in the average daily values of ambient temperature and wind speed are shown in Fig. 2. The average daily values of the ambient temperature reached the values in the range of 285.5–299.6 K (during the sunny hours from the range of 286.7–304 K) in July and 289.6–297.9 K (during the sunny hours from the range of 291.7–300.7 K) in August. The mean values of wind speed were in the range of 0.20–0.45 m/s (during the sunny hours from the range of 0.09–0.75 m/s) in July and 0–0.54 m/s (during the sunny hours from the range of 0–0.86 m/s) in August.

**Fig. 3:**
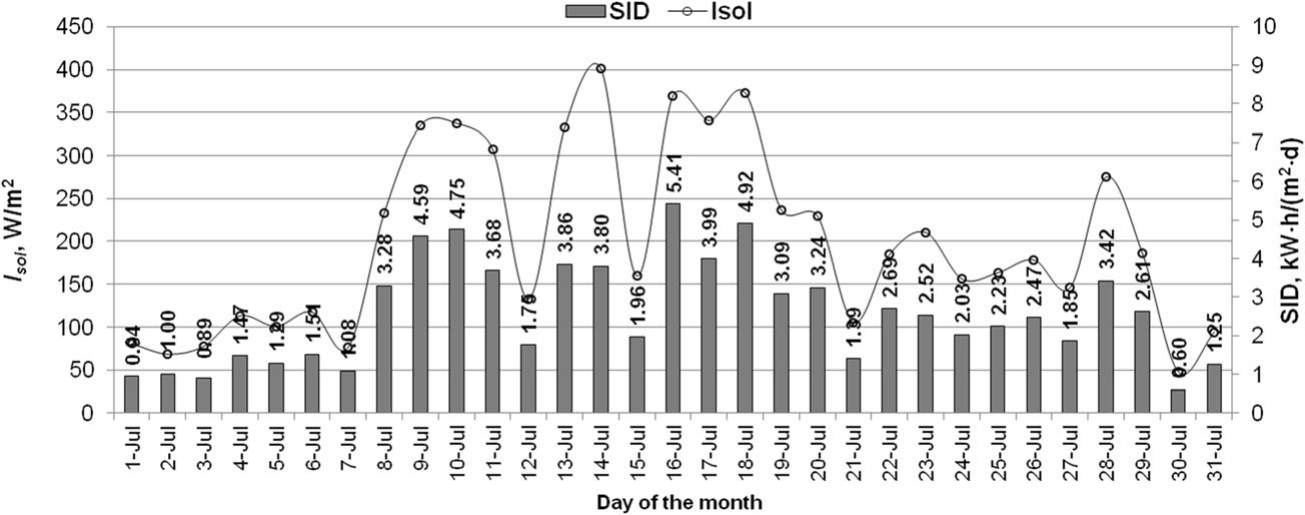
Solar irradiance and the solar irradiation per day variations in July

**Fig. 4:**
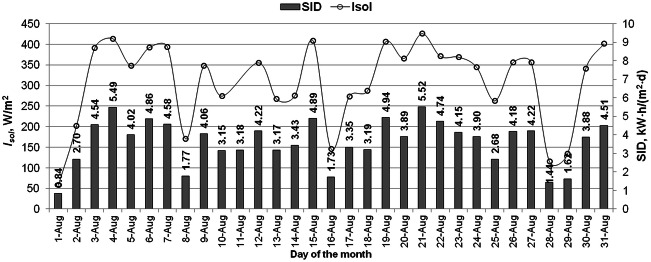
Solar irradiance and the solar irradiation per day variations in August

The average values of the solar irradiance and the solar irradiation per day (*SID*) are shown in Figs. 3 and 4. The average values of solar irradiance for individual days ranged between 48 W/m^2^ and 426 W/m^2^. The highest value of the solar irradiation per day was recorded on August 21 and reached 5.52 kWh/(m^2^·d), whereas the lowest value was recorded on July 30 and was equal to 0.60 kWh/(m^2^·d). The average solar irradiation per day was set at 2.6 kWh/m^2^ for July and 3.64 kWh/m^2^ for August. In July, only 12 days with a *SID* above 3 kWh/m^2^ were recorded and in August 24 days with a *SID* above 3 kWh/m^2^. The solar irradiation per month for July reached 80 kWh/m^2^, and for August, it amounted to 112.8 kWh/m^2^.

**Fig. 5:**
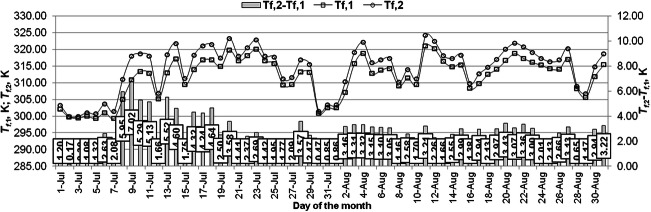
Values of inlet and outlet fluid temperature

The average daily glycol temperature at the inlet to the collector ranged from 299.2 to 321 K and at the collector outlet reached values in the range of 299.9 K–324.2 K in the period considered. The maximum value of the working fluid temperature difference at the outlet and inlet to the solar collector was 7 K (Fig. 5).

**Fig. 6:**
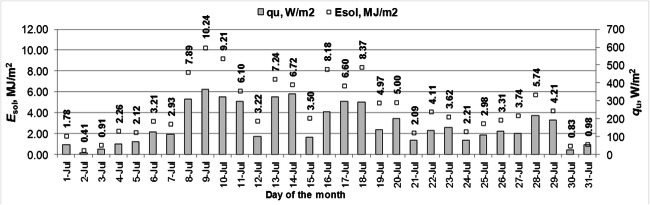
The useful heat gain from the solar collector and the energy absorbed by 1 m^2^ of the solar collector surface in July

**Fig. 7:**
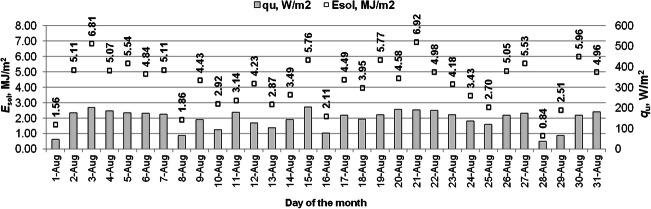
The useful heat gain from the solar collector and the energy absorbed by 1 m^2^ of the solar collector surface in August

The average values of the useful heat gain by the working fluid and the energy absorbed by 1 m^2^ of the solar collector surface are presented in Figs. 6 and 7. The average thermal gain was found to be 163 W/m^2^ (587 W) in July and 145 W/m^2^ (522 W) in August, respectively. The unit energy yield in the solar collector was determined for individual days from 0.41 to 10.24 MJ/(m^2^·d) in July and from 0.84 to 6.92 MJ/(m^2^·d) in August. In July 135 MJ/m^2^ (135 kWh/month) thermal energy was obtained, and 131 MJ/m^2^ (131 kWh/month) in August.

**Fig. 8:**
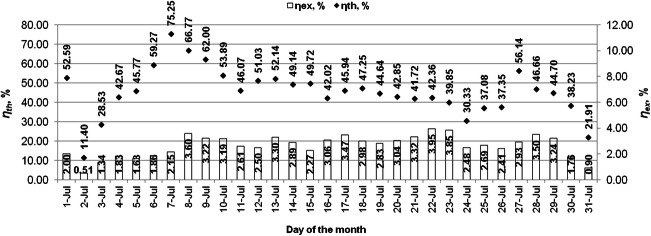
Daily averages of thermal and exergy efficiencies of the solar collector in July

**Fig. 9:**
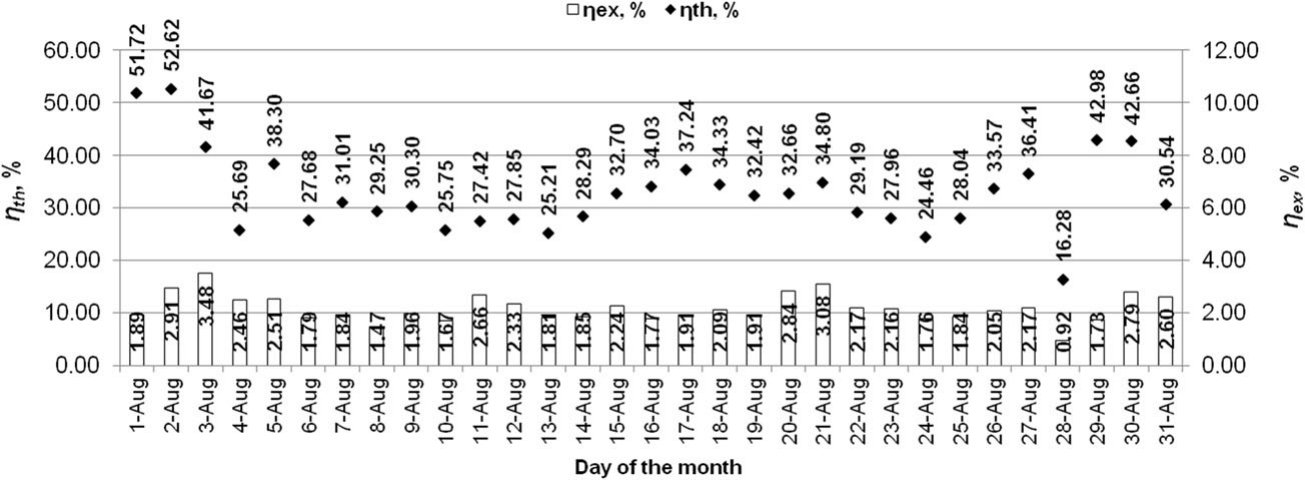
Daily averages of thermal and exergy efficiencies of the solar collector in August

The average daily values of the energy efficiency and exergy efficiency characterizing the evacuated tube solar collector are shown in Figs. 8 and 9. The average daily thermal efficiencies of the solar collector were obtained reaching 11.40–75.25% in July and 16.28–52.62% in August. The average monthly energy efficiency of the solar collector in July and August was 45.3% and 32.9%, respectively. The average daily exergy efficiencies of the collector in July reached the values from 0.51 to 3.95% and in August from 0.92 to 3.48%. The average monthly exergy efficiency of the solar collector was 2.62% in July and 2.15% in August.

Despite less favorable weather conditions, higher thermal efficiency of the solar collector was obtained in July. The evacuated solar collectors type heat pipe operate efficiently even in low sunlight and at low outdoor air temperature. The smaller the difference between the fluid inlet temperature to the collector and the ambient temperature is, the greater the energy efficiency of the collector. In the case of August, for a difference of the fluid temperature at the collector inlet and an ambient temperature of 10.1 K, the collector’s thermal efficiency was 52%. For July, the collector’s thermal efficiency of 75% was obtained with a difference of these temperatures amounting to 7 K. The days in which the collector worked in July with the highest efficiency were characterized by low irradiance (solar radiation intensity up to 200 W/m^2^) and a relatively low temperature difference of outlet and inlet to the collector equal to 2 K.

The studies on the solar collector performance under real conditions require a lot of measurements to be carried out while maintaining the same reference conditions. The thermal and exergy efficiencies of the solar collector are determined by weather conditions that change over time, such as solar radiation intensity, ambient temperature, and wind speed, as well as exploitation parameters: temperature for working fluid flowing into the collector and working fluid volume flow.

**Fig. 10:**
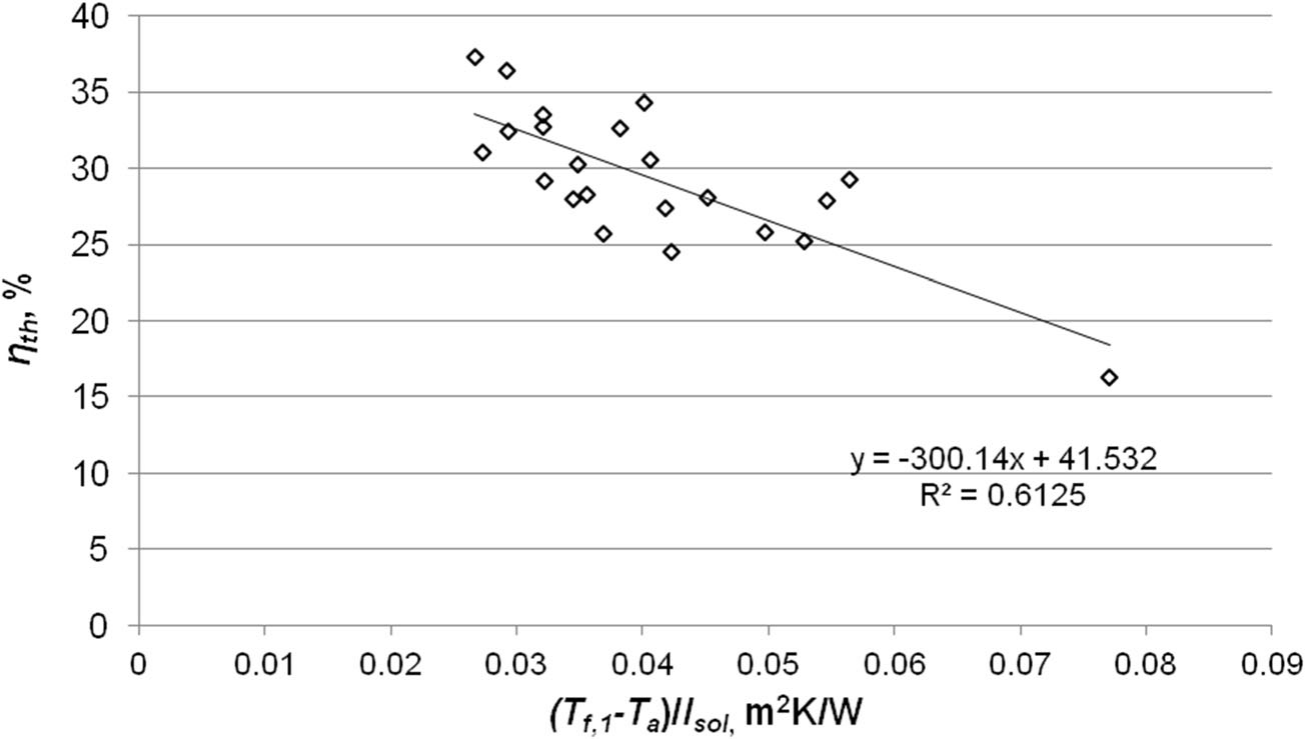
Variations of energy efficiency of the solar collector versus (*T*_*f,* 1_-*T*_*a*_)/*I*_*sol*_

Figure 10 presents the dependence of the collector thermal efficiency on the reduced temperature difference, based on the data for August. The value of the determination factor *R*^2^ = 0.6125 (*R* = 0.7826) indicates a strong correlation relationship between both parameters. The intersection point of the thermal efficiency characteristics with the y-axis approximately determines the optical efficiency of the solar collector, more precisely, the product *F*_*R*_·(*τ·α*) = 0.42. The tangent of the inclination angle of the efficiency curve to the x-axis corresponds to the product *F*_*R*_·*U*_*L*_. It determines the heat losses associated with the construction and material parameters of the collector. The smaller the angle of inclination is, the lower the heat loss.

**Fig. 11:**
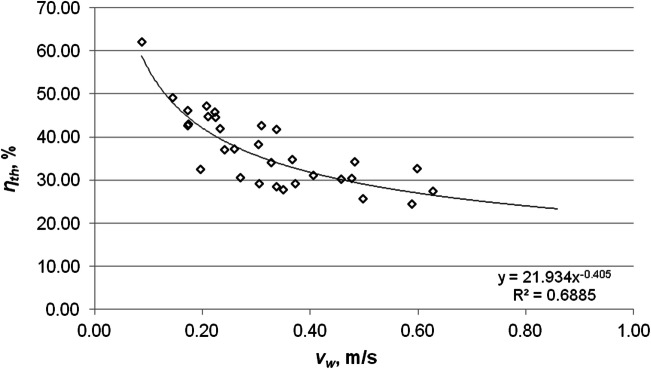
Variations of the thermal efficiency of the solar collector versus the wind speed

**Fig. 12:**
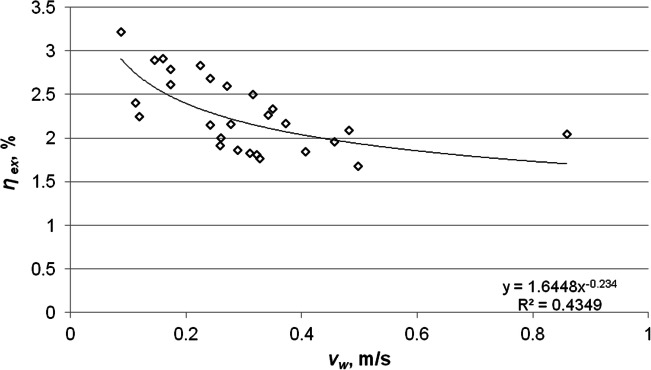
Variations of the exergy efficiency of the solar collector versus the wind speed

The analysis pertaining to the impact of the wind speed on the collector’s thermal and exergy efficiencies was based on daily average values, but took into account only those hours in which the solar collector worked effectively. The anemometer was placed near the collector, on a bracket moved away from the outer wall. In the considered period, the average wind speed did not exceed 0.9 m/s. Both the collector’s thermal efficiency and exergy efficiency decrease as the wind speed increases. Figures 11 and 12 show the variations of the energy and exergy efficiencies versus the wind speed. The value of the determination factor *R*^2^ = 0.6885 (*R* = 0.8298) indicates a strong correlation relationship between thermal efficiency and the wind speed. In the case of dependence of exergy efficiency on the wind speed, the coefficient of determination was obtained at *R*^2^ = 0.4349 (*R* = 0.6595), which means a weaker relationship in these parameters compared to the correlation of the thermal efficiency and the wind speed. Increasing the wind speed from 0.09 to 0.86 m/s decreases the thermal efficiency and the exergy efficiency by 67% and by 41%, respectively.

## Conclusions

In the analysis of the thermal performance of the evacuated tube solar collector, the influence of real variable weather (i.e., solar irradiance, ambient temperature, wind speed) and operating conditions (working fluid temperature at the inlet and outlet of the collector) was taken into account.

The average value of the solar irradiation per day for the considered time period (2 months) was 3.1 kWh/(m^2^·d), while the mean value of the ambient temperature was 292.6 K. The average values of the useful heat gain from the solar collector in July and August reached 163 W/m^2^ and 145 W/m^2^, respectively. The average monthly thermal yield for the solar collector was 478.8 MJ/month.

The average monthly energy efficiency of the heat pipe evacuated tube solar collector in July and August were 45.3% and 32.9%, respectively. The average monthly exergy efficiency of the solar collector amounted to 2.62% in July and 2.15% in August.

It was found that an increase in the temperature difference between the mixture of water and propylene glycol at the solar collector inlet and ambient temperature causes a slight increase in the exergy efficiency and a decrease in the thermal efficiency. This conclusion is consistent with the results of the experimental investigations carried out by Jafarkazemi et al. (2016).

The increase in the wind velocity contributes to the decrease in both energy and exergy efficiency of the evacuated tube solar collector.

## Nomenclature

*A*: area (m^2^)
*c*_*p*_: specific heat capacity at constant pressure (J/(kg·K))
$$ \dot{E}x $$: exergy rate (W)
*F*_*R*_: collector heat removal factor
*I*: radiation intensity (W/m^2^)
$\dot{m}$: fluid mass-flow rate (kg/s)
$\dot{Q}$: heat energy rate (W)
*SID*: solar irradiation per day (kWh/(m^2^·d))
*T*: temperature (K)
*U*_*L*_: overall heat loss coefficient of the solar collector (W/(m^2^·K))

## Greek symbols

*η*: instantaneous efficiency
τ: transmittance of the glass cover
α: absorptance of the absorber plate

## Subscripts

a: ambient
col: collector
EX: exergy
m: mean
sol: solar
TH: thermal
u: useful
W-PG: mixture of water and propylene glycol (50%/50%)
f: circulated fluid
1: inlet
2: outlet”

## References

[CR1] Alfaro-Ayala JA, Martínez-Rodríguez G, Picón-Núńez M, Uribe-Ramírez AR, Gallegos-Muńoz A (2015). Numerical study of a low temperature water-in-glass evacuated tube solar collector. Energy Convers Manag.

[CR2] Allouhi A, Amine MB, Buker MS, Kousksou T, Jamil A (2019) Forced-circulation solar water heating system using heat pipe-flat plate collectors: energy and exergy analysis. Energy. 10.1016/j.energy.2019.05.063

[CR3] Ataee S, Ameri M (2015). Energy and exergy analysis of all-glass evacuated solar collector tubes with coaxial fluid conduit. Sol Energy.

[CR4] Ayompe LM, Duffy A (2013). Thermal performance analysis of a solar water heating system with heat pipe evacuated tube collector using data from a field trial. Sol Energy.

[CR5] Ayompe LM, Duffy A, Mc Keever M, Conlon M, Mc Cormack SJ (2011). Comparative field performance study of flat-plate and heat pipe evacuated tube collector in a temperate climate. Energy.

[CR6] Chekerovska M, Filkoski RV (2015). Efficiency of liquid flat-plate solar energy collector with solar tracking system. Therm Sci.

[CR7] Chopra K, Tyagi VV, Pandey AK, Sari A (2018). Global advancement on experimental and thermal analysis of evacuated tube collector with and without heat pipe systems and possible applications. Appl Energy.

[CR8] Chwieduk D (2011). Building’s solar power engineering.

[CR9] Daghigh R, Shafieian A (2016). Theoretical and experimental analysis of thermal performance of a solar water heating system with evacuated tube heat pipe collector. Appl Therm Eng.

[CR10] Elsheniti MB, Kotb A, Elsamni O (2019). Thermal performance of a heat-pipe evacuated-tube solar collector at high inlet temperatures. Appl Therm Eng.

[CR11] Farahat S, Sarhaddi F, Ajam H (2009). Exergetic optimization of flat plate solar collectors. Renew Energy.

[CR12] Hayek M, Assaf J, Lteif W (2011). Experimental investigation of the performance of evacuated-tube solar collectors under eastern Mediterranean climatic conditions. Energy Procedia.

[CR13] Jafarkazemi F, Ahmadifard E (2013). Energetic and exergetic evaluation of flat plate solar collectors. Renew Energy.

[CR14] Jafarkazemi F, Ahmadifard E, Abdi H (2016). Energy and exergy efficiency of heat pipe evacuated tube solar collectors. Therm Sci.

[CR15] Joo H-J, Kwak H-Y (2017). Experimental analysis of thermal performance according to heat pipe working fluids for evacuated tube solar collector. Heat Mass Transf.

[CR16] Kalogirou SA, Karellas S, Braimakis K, Stanciu C, Badescu V (2016). Exergy analysis of solar thermal collectors and processes. Prog Energy Combust Sci.

[CR17] Kaya H, Arslan K (2019). Numerical investigation of efficiency and economic analysis of an evacuated U-tube solar collector with different nanofluids. Heat Mass Transf.

[CR18] Kaya H, Arslan K, Eltugral N (2018). Experimental investigation of thermal performance of an evacuated U-tube solar collector with ZnO/etylene glycol-pure water nanofluids. Renew Energy.

[CR19] Krawczyk DA, Żukowski M, Rodero A (2019). Efficiency of a solar collector system for the public building depending on its location. Environ Sci Pollut Res.

[CR20] Kumar SS, Kumar KM, Kumar SRS (2017). Design of evacuated tube solar collector with heat pipe. Materials Today: Proceedings.

[CR21] Maraj A, Londo A, Gebremedhin A, Firat C (2019). Energy performance analysis of a forced circulation solar water heating system equipped with a heat pipe evacuated tube collector under the Mediterranean climate conditions. Renew Energy.

[CR22] Naik BK, Bhowmik M, Muthukumar P (2019). Experimental investigation and numerical modelling on the performance assessments of evacuated U-tube solar collector systems. Renew Energy.

[CR23] Nkwetta DN, Smyth M, Haghighat F, Zacharopoulos A, Hyde T (2013). Experimental performance evaluation and comparative analyses of heat pipe and direct flow augmented solar collectors. Appl Therm Eng.

[CR24] Park SR, Pandey AK, Tyagi VV, Tyagi SK (2014). Energy and exergy analysis of typical renewable energy systems. Renew Sust Energ Rev.

[CR25] Petela R (1964). Exergy of heat radiation. ASME Journal of Heat Transfer.

[CR26] Petela K (2017) Definition and analysis of the exergy efficiency of a solar collector. In: Werle S (ed) environmental engineering in energy and automotive, Institute of Thermal Technology Silesian University of technology Gliwice, pp 151-168 (in Polish)

[CR27] Sabiha MA, Saidur R, Mekhilef S, Mahian O (2015). Progress and latest developments of evacuated tube solar collectors. Renew Sust Energ Rev.

[CR28] Saxena G, Gaur MK (2018). Exergy analysis of evacuated tube solar collectors: a review. Int J Exergy.

[CR29] Shafieian A, Khiadani M, Nosrati A (2019). Thermal performance of an evacuated tube heat pipe solar water heating system in cold season. Appl Therm Eng.

[CR30] Sharafeldin MA, Gróf G, Abu-Nada E, Mahian O (2019). Evacuated tube solar collector performance using copper nanofluid: energy and environmental analysis. Appl Therm Eng.

[CR31] Sharma N, Diaz G (2011). Performance model of a novel evacuated-tube solar collector based on mini channels. Sol Energy.

[CR32] Siuta-Olcha A (2012) Experimental and theoretical studies of a hot water tank with thermal stratification. Monographs Vol. 103, Polish Academy of Sciences, the Committee of Environmental Engineering, Lublin (in Polish)

[CR33] Siuta-Olcha A, Cholewa T, Dopieralska-Howoruszko K (2019). Experimental investigations of energy and exergy efficiencies of an evacuated tube solar collector. Proceedings.

[CR34] Szargut J, Petela R (1965). Exergy.

[CR35] Tong Y, Kim HM, Cho HH (2016). Theoretical investigation of the thermal performance of evacuated heat pipe solar collector with optimum tilt angle under various operating conditions. J Mech Sci Technol.

[CR36] Weiss W, Spörk-Dür M (2018) Solar heat worldwide global market development and trends in 2017/detailed market figures 2016. IEA-SHCP. http://www.iea-shc.org/data/sites/1/publications/Solar-Heat-Worldwide-2018.pdf. Accessed 29 April 2019

[CR37] Zambolin E, Del Col D (2010). Experimental analysis of thermal performance of flat plate and evacuated tube solar collectors in stationary standard and daily conditions. Sol Energy.

